# A betabaculovirus encoding a *gp64* homolog

**DOI:** 10.1186/s12864-016-2408-9

**Published:** 2016-02-04

**Authors:** Daniel M P. Ardisson-Araújo, Bruna T. Pereira, Fernando L. Melo, Bergmann M. Ribeiro, Sônia N. Báo, Paolo M. de A. Zanotto, Flávio Moscardi, Elliot W. Kitajima, Daniel R. Sosa-Gomez, José L. C. Wolff

**Affiliations:** Departamento de Biologia Celular, Instituto de Ciências Biológicas, Universidade de Brasília, Brasília, DF Brazil; Programa de Pós-graduação Interunidades em Biotecnologia, Instituto de Ciências Biomédicas (ICB), Universidade de São Paulo (USP), São Paulo, São Paulo Brazil; Laboratório de Evolução Molecular e Bioinformática (LEMB-ICB), Universidade de São Paulo, São Paulo, SP Brazil; Empresa Brasileira de Pesquisa Agropecuária, Centro Nacional de Pesquisa de Soja, Londrina, Paraná PR Brazil; NAP/MEPA, Departamento de Fitopatologia e Nematologia, ESALQ, Universidade de São Paulo, Piracicaba, SP Brazil; Laboratório de Biologia Molecular e Virologia, Centro de Ciências Biológicas e da Saúde (CCBS), Universidade Presbiteriana Mackenzie, São Paulo, SP Brazil

## Abstract

**Background:**

A betabaculovirus (DisaGV) was isolated from *Diatraea saccharalis* (Lepidoptera: Crambidae), one of the most important insect pests of the sugarcane and other monocot cultures in Brazil.

**Results:**

The complete genome sequence of DisaGV was determined using the 454-pyrosequencing method. The genome was 98,392 bp long, which makes it the smallest lepidopteran-infecting baculovirus sequenced to date. It had a G + C content of 29.7 % encoding 125 putative open reading frames (ORF). All the 37 baculovirus core genes and a set of 19 betabaculovirus-specific genes were found. A group of 13 putative genes was not found in any other baculovirus genome sequenced so far. A phylogenetic analysis indicated that DisaGV is a member of *Betabaculovirus* genus and that it is a sister group to a cluster formed by ChocGV, ErelGV, PiraGV isolates, ClanGV, CaLGV, CpGV, CrleGV, AdorGV, PhopGV and EpapGV. Surprisingly, we found in the DisaGV genome a G protein-coupled receptor related to lepidopteran and other insect virus genes and a *gp64* homolog, which is likely a product of horizontal gene transfer from Group 1 alphabaculoviruses.

**Conclusion:**

DisaGV represents a distinct lineage of the genus *Betabaculovirus*. It is closely related to the CpGV-related group and presents the smallest genome in size so far. Remarkably, we found a homolog of *gp64*, which was reported solely in group 1 alphabaculovirus genomes so far.

**Electronic supplementary material:**

The online version of this article (doi:10.1186/s12864-016-2408-9) contains supplementary material, which is available to authorized users.

## Background

Brazil is the largest sugarcane (*Saccharum officinarum*, L.) and bioethanol producer in the world [1, 2]. Nowadays, sugarcane is grown on an area over 8 million hectares for both sugar and alcohol production [2]. As with other cultures cultivated over large areas, pest control is of crucial importance. The sugarcane borer *Diatraea saccharalis* Fabr. (Lepidoptera: Crambidae) is present in all sugarcane-producing regions of the country, and is considered the major sugarcane pest, especially in the Southeast region [3]. Biological control based on the release of the parasitoid *Cotesia flavipes* (Cameron) (Hymenoptera: Braconidae) has been used with success in the control of the sugarcane borer [4, 5]. However, other complementary and compatible methods, such as the application of baculoviruses, would be highly desirable.

Baculoviruses are a large group of insect-specific viruses with circular double-stranded DNA, whose hallmark is the presence of occlusion bodies (OBs) [6]. The family *Baculoviridae* comprises four genera: two of them, *Alphabaculovirus* and *Betabaculovirus*, infect insects of the order Lepidoptera; the other two *Gammabaculovirus* and *Deltabaculovirus* infect insects of the orders Hymenoptera and Diptera [7] respectively. To date, the genomes of 73 baculovirus species were completely sequenced, and 17 of them are betabaculoviruses.

The baculovirus from the species *Anticarsia gemmatalis multiple nucleopolyhedrovirus* (AgMNPV) has been used in Brazil in one of the largest biocontrol programs in the world to control an insect pest [8]. Other successful programs with baculoviruses have been reported elsewhere in the world [6]. The success of the AgMNPV program is due to a combination of factors, such as: high virulence, dead larvae can be collected directly from the field to be used as inoculum, efficient application technology, etc. Nevertheless, development is needed on pest species that are not so easily exposed to the virus, as in the case of borers. Large-scale DNA sequencing provides information on complete viral genomes allowing for “omic” approaches that will eventually facilitate the development of application strategies. Since Brazil has high biodiversity, several baculoviruses have been found and their genomes sequenced [9–14]. With this prospect in mind, we have sequenced and analyzed the genome of a Diatraea saccharalis granulovirus (DisaGV), the first betabaculovirus isolated from a member of the family Crambidae. The presence of a functional *gp64* homolog [15] in a betabaculovirus was a unique and remarkable finding.

## Results and discussion

### Viral infection confirmation

**Fig. 1:**
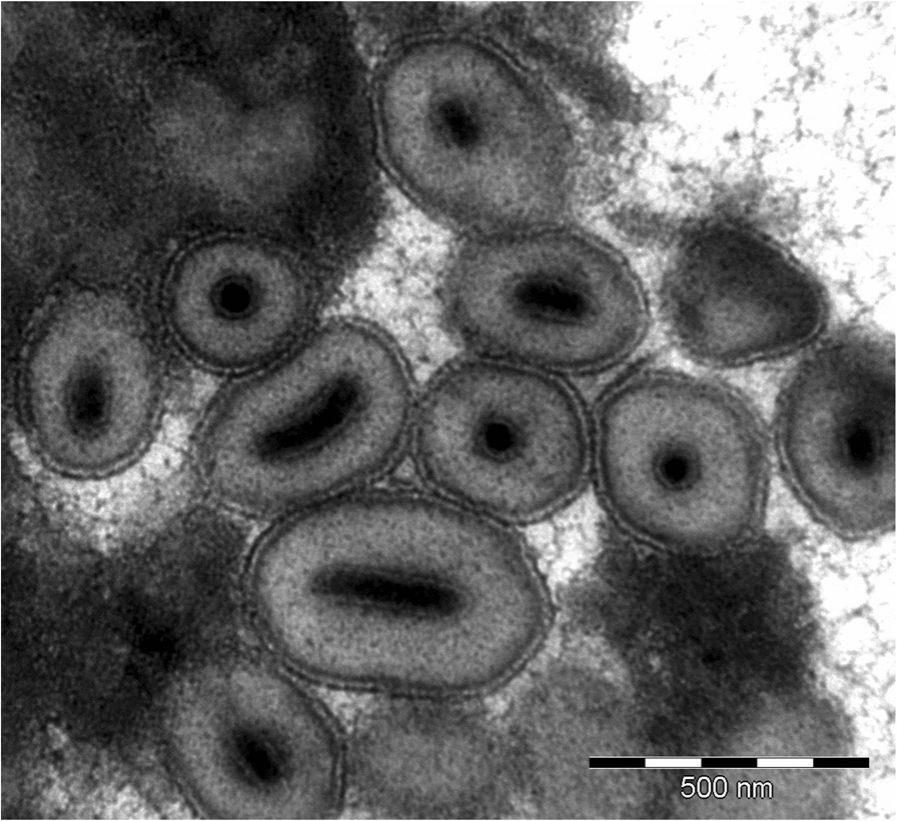
Ultrastructural analysis of Diatraea saccharalis granulovirus (DisaGV). Transmission electron micrograph reveals granular occlusion bodies containing singly embedded rod-shaped nucleocapsid (scale bars = 0.5 μm)

Larvae of *Diatraea saccharalis* with virus infection symptoms were found in sugarcane fields in the Southern Brazil. We performed the structural characterization of the putative virus and a granulovirus infection was confirmed by transmission electron microscopy of OBs extracted from larvae cadavers. Each elliptical granule had a single rod-shaped virion surrounded by a robust protein matrix coat (Fig. 1), indicating the typical morphology of granuloviruses (GVs) [6]. Since the protein matrix is formed by granulin produced in large amounts during late infection and because it is highly conserved among lepidopteran-infective baculovirus, we amplified and sequenced the *granulin* gene in order to obtain an initial confirmation to the viral type (data not shown). The 747 bp length of the DisaGV *granulin* had high amino acid identity with orthologs from the genus *Betabaculovirus* (data not shown). Importantly, a granulovirus was previously isolated from *D. saccharalis* caterpillars sampled from sugarcane crops in the southern United States. However, there is no available sequence to establish any phylogenetic relationship to the Brazilian strain described here [16].

### DisaGV genome and phylogeny

**Fig. 2:**
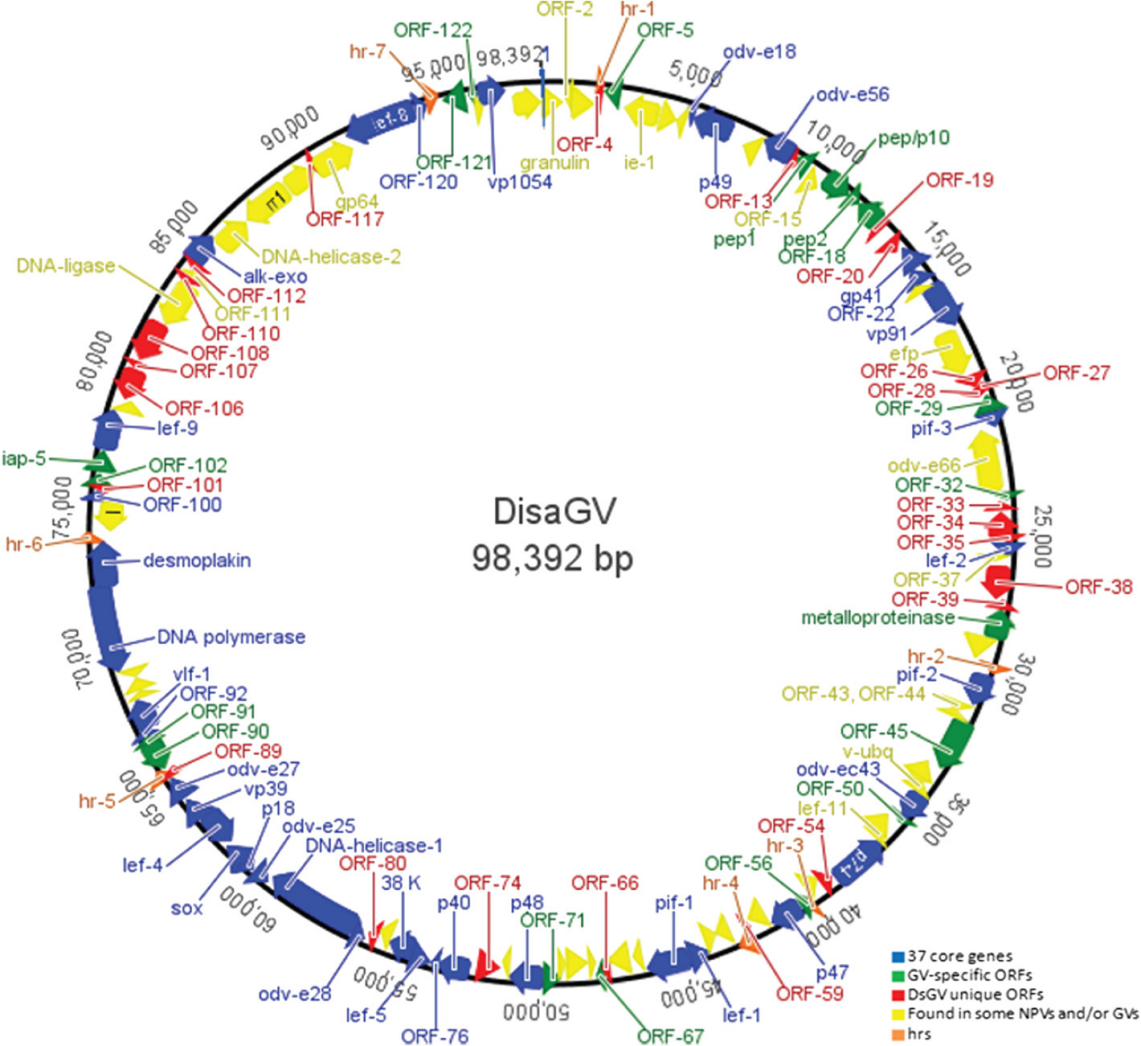
Circular genome map of DisaGV with all genes identified on the 98,392 bp long. Arrows show the transcripcional orientation and relative size of each ORF. Those are colored according to their presence into baculovirus genera: in blue the 37 core genes, in green only betabaculovirus-specific genes, in red the DisaGV unique genes, in yellow genes found in some subjects of both alpha and betabaculovirus, and homologous regions (hrs) in orange

**Fig. 3:**
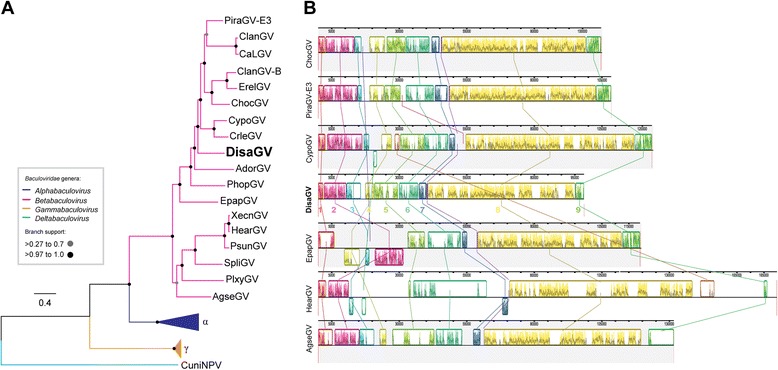
Maximum-likelihood tree for *Betabaculovirus* and genome comparison. **a** The phylogeny was based on the concatenated amino acid sequences of the 37 core proteins identified in all baculovirus genome completely sequenced so far (Additional file 2: Table S2). We collapsed gammabaculoviruses (orange, γ) and alphaphabaculoviruses (dark blue, α). The CuniNPV was used as root (light blue). DisaGV (boldface) is a betabaculovirus and sister species of the cluster formed by CpGV-related species. **b** Genome comparison of the DisaGV genome against some related species including AgseGV, ChocGV, CpGV, EpapGV, and ErelGV. Locally collinear blocks (LCB) are numbered in the DisaGV genome from 1 to 9. Same colors depict same LCBs across the genomes. Rearrangement can be seen among the species

The complete genome of DisaGV (Genbank accession number: KP296186) was 98,392 bp in length (mean coverage of 36 x), which makes the DisaGV the smallest betabaculovirus sequenced to date, followed by Adoxophyes orana granulovirus (AdorGV) (99,657 bp) [17] and *Plutella xylostella granulovirus* (PlxyGV) (100,999 bp) [18]. The G + C content was 29.7 %, a typically low value found among GVs, and potentially encoded 125 open reading frames (ORFs) with at least 50 predicted amino acid residues (Additional file 1: Table S1 and Fig. 2). The current baculovirus proposed species demarcation criterion is based on pairwise nucleotide distances estimated using the Kimura 2-parameter model of nucleotide substitution for three genes, *granulin*, *lef-8*, and *lef-9* [7]. The pairwise distances of the viral sequences of DisaGV to other betabaculoviruses for both single loci and concatenated alignment are well in excess of 0.05 substitutions/site, fulfilling all the criteria for a novel species (data not shown). In order to investigate the phylogenetic relationship of DisaGV to other baculoviruses, we carried out a maximum likelihood phylogenetic analysis based on the alignment of the 37 baculovirus core proteins from all baculovirus genomes publicly available using solely the unique species (Additional file 2: Table S2). In agreement with both OB ultrastructural analysis and *granulin* gene sequencing (data not shown), we found DisaGV as sister taxa of the cluster formed by Choristoneura occidentalis granulovirus (ChocGV), Erinnyis ello granulovirus (ErelGV), Pieris rapae granulovirus (PiraGV) isolates, Clostera anachoreta granulovirus (ClanGV), Clostera anastomosis granulovirus (CaLGV), Cydia pomonella granulovirus (CpGV) and Cryptophlebia leucotreta granulovirus (CrleGV) (Fig. 3a).

Moreover, we performed a genomic comparison among some selected betabaculovirus genomes by MAUVE analysis. We found nine Locally Collinear Blocks (LCB), composed of genomic segments that appear to have the same relative position of their shared genes (Fig. 3b). Interestingly, LCB5 (from bp 20013 to 37032), LCB7 (from bp 40326 to 76348) and LCB8 (from bp 76601 to 87652) had an unexpected gene content composition. LCB5 lacked baculovirus core genes (*χ*^2^ = 2.46, *p* < 0.05, df =3), while LCB7 had a higher than expected number (*χ*^2^ = 3.84, *p* < 0.05, df =3) and LCB8 had a higher than expected number of DisaGV-unique genes, a lower than expected number of baculovirus core genes and less than expected GV-specific genes (*χ*^2^ = 5.12, *p* < 0.01, df =3).

DisaGV had a *dna*-*ligase* (*disa107*) and two *helicase* genes (*helicase-1*, *disa081* and *helicase-2*, *disa111*) probably involved in replication, repair, and recombination of DNA [19]. We also identified a *deoxyuridine triphosphatase* (*dut*) gene (*disa073*) and the *ribonucleotide reductase* subunits *rr1* (*disa112*) and *rr2a* (*disa113*), involved in nucleotide metabolism. Nevertheless, the role of those genes during baculovirus infection is not clear. It was noteworthy the absence of several genes for early transcription factors, such as the *ie-0*, *ie-2*, and *pe38*. There were also no similar sequences to the *baculovirus repeated ORFs* (*bro* genes), to the *ecdysteroid UDP-glucosyltransferase* (*egt*), to the apoptosis inhibitor *p35*, and also to the *cathepsin* and *chitinase* genes. We observed that the *egt* gene was absent only in the genomes of four other GVs, Helicoverpa armigera granulovirus (HearGV), Pseudaletia unipuncta granulovirus (PsunGV), Spodoptera litura granulovirus isolate K1 (SpliGV-K1) and Xestia c-nigrum granulovirus (XecnGV), that form a distinct phylogenic cluster. On the other hand, the *p35* gene was found only in the genomes of ChocGV, CaLGV, ClanGV (Data not shown). The absence of the *cathepsin* and *chitinase* genes may be compensated by the presence of the putative gene for *matrix metalloproteinase* (a *stromelysin-1-like* gene, *disa040*). Whereas the loss of the *cathepsin* and *chitinase* genes is a common event among the betabaculoviruses [10], the *matrix metalloproteinase* gene is present in all betabaculoviruses sequenced to date [20]. The expression of a functional CpGV-encoded metalloproteinase into the Autographa californica multiple nucleopolyhedrovirus (AcMNPV) genome enhanced the virus virulence, promoted larval melanization, and could partially substitute for the viral cathepsin [21].

### DisaGV unique genes

**Fig. 4:**
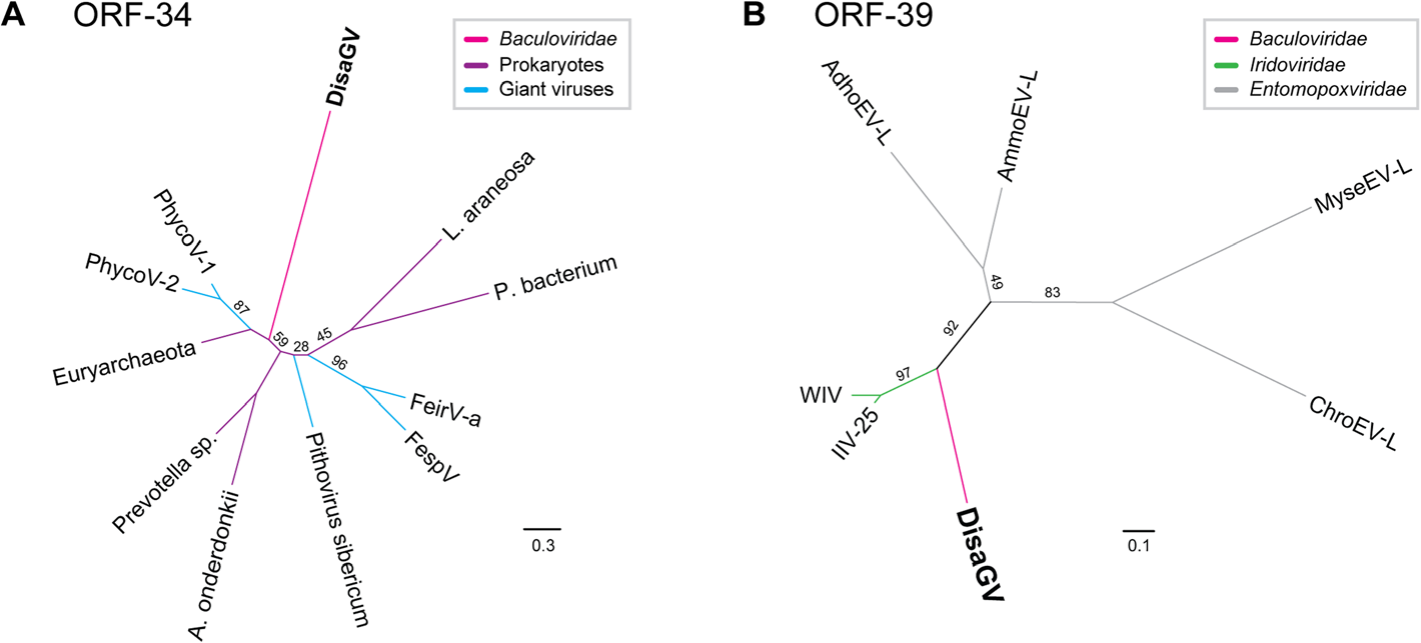
Maximum likelihood phylogenetic trees of both Disa034 (**a**) and Disa039 (**b**) based on their predicted amino acid sequence. We used the RaxML method under the LG + I + G model for Disa034 and WAG + I + F for Disa039 with a nonparametric bootstrap to support the branches. Organisms: (**a**) *Organic Lake phycodnaviruses* (PhycoV-1 and PhycoV-2), *Feldmannia species virus* (FespV), *Feldmannia irregularis virus a* (FeirV-a) and Prokaryotes. **b** *Wiseana iridescent virus* (WIV), Invertebrate iridovirus 25 (IIV-25), *Amsacta moorei entomopoxvirus ‘L’* (AmmoEV-L), Adoxophyes honmai entomopoxvirus ‘L’ (AdhoEV-L), Mythimna separata entomopoxvirus ‘L’ (MyseEV-L) and Choristoneura rosaceana entomopoxvirus ‘L’ (ChroEV-L)

Homologs to 25 DisaGV ORFs were not found in the genome of other baculoviruses. Taking into account the 450 bp region upstream of each unique ORF, three of them presented no previously characterized promoter motifs, 12 contained exclusively early promoter motifs (TATAW, TATAWAW, TATAWTW with W = A or T), and ten had both early and late (A/TTAAG) motifs (Additional file 1: Table S1). Two unique ORFs, *disa034* and *disa039* showed significant BlastP hits to other dsDNA virus sequences publicly available. ORF *disa034* encoded a putative 310 aa protein that showed 26 % amino acid identity (e-value = 1e-06) to a 247 aa length protein of a phycodnavirus (*Feldmannia irregularis virus a*, AAR26869) (Fig. 4a). By motif search using the Pfam database [22], we found a conserved domain related to a PD-(D/E)XK nuclease superfamily, a highly diverse class of proteins. The exact biological functions, substrate specificity and molecular mechanisms of reactions for those nucleases remain unknown. However, quite a few enzymes are thought to be related to many cell process including replication, restriction, DNA repair and tRNA–intron splicing [23]. Moreover, ORF *disa039* coded for a hypothetical protein related to insect-infecting dsDNA viruses including *Wiseana iridescent virus* (WIV) (YP_004732905, 131 aa) and *Amsacta moorei entomopoxvirus ‘L’* (NP_064857, 158 aa) (Fig. 4b). Phycodnaviruses are eukaryotic algae viruses and seem to share a common ancestor with other insect dsDNA viruses, including iridoviruses and entomopoxviruses, which share baculovirus genes as well. Several baculovirus genes were found into the genome of those viruses, suggesting the occurrence of lateral gene transfer during co-infection in the same insect host, as probably expected to *disa034* and *disa039* [24].

### G protein-coupled receptor (GPCR)

**Fig. 5:**
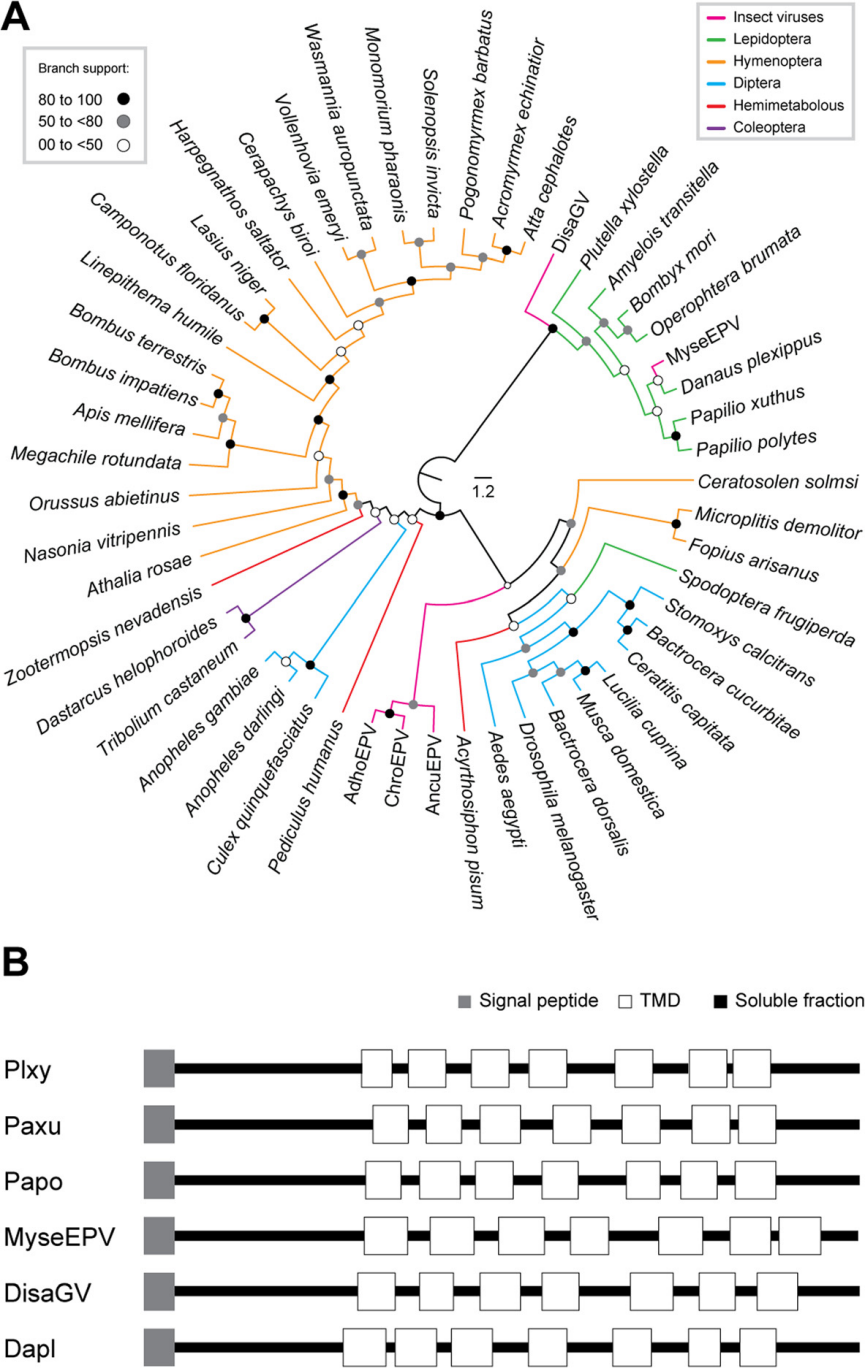
*In silico* analyses of Disa038, a betabaculovirus-encoded G protein-coupled receptor gene. **a** Phylogenetic analysis of selected arthropod GPCRs. Disa038 sequence clustered with lepidopteran and an entomopoxvirus proteins. We performed the RaxML method under the WAG + I + G + F model with a nonparametric bootstrap. The tree is presented as a cladogram. **b** Schematic representation of Disa038 and phylogenetically related proteins. The betabaculovirus GPCR retained all the structures observed in the homologs including the signal peptide (gray), soluble fraction (black), and the transmembrane domains (TMDs, white). Plxy, *Plutella xylostella*; Psxu, *Papilio xuthus*; Papo, *Papilio polytes*; MyseEV, Mythimna separata entomopoxvirus ‘L’; and Dapl, *Danaus plexippus*

We also found another unique ORF (*disa038*) encoding a polypeptide related to a putative class B secretin-like G-protein coupled receptor (GPCR) of lepidopteran and an entomopoxvirus (Fig. 5a). GPCRs are cell membrane-associated GTPases that transmit signals from the environment to the cell inside or between cells. This allows them to react to a corresponding variety of extracellular stimuli that can be mediated by different peptides, lipids, proteins, nucleotides, nucleosides, organic odorants and photons [25]. This type of receptor has been described in many animal species despite not being quite common in virus genomes. We found a predicted signal peptide and seven trans-membrane domains in Disa038 (Fig. 5b), which strongly suggests that it is a member of the Secretin family [26]. Three subfamilies are recognized for this family and one of them, the B2 contains receptors with long extracellular N-termini as observed for both the predicted Disa038 and the other related proteins. It is not clear what role of this gene plays in the DisaGV life cycle. Nevertheless, it is noteworthy that human herpesvirus uses virally encoded GPCR to hijack cellular signaling network, which suggests a similar mechanism in DisaGV [27].

### GP64

**Fig. 6:**
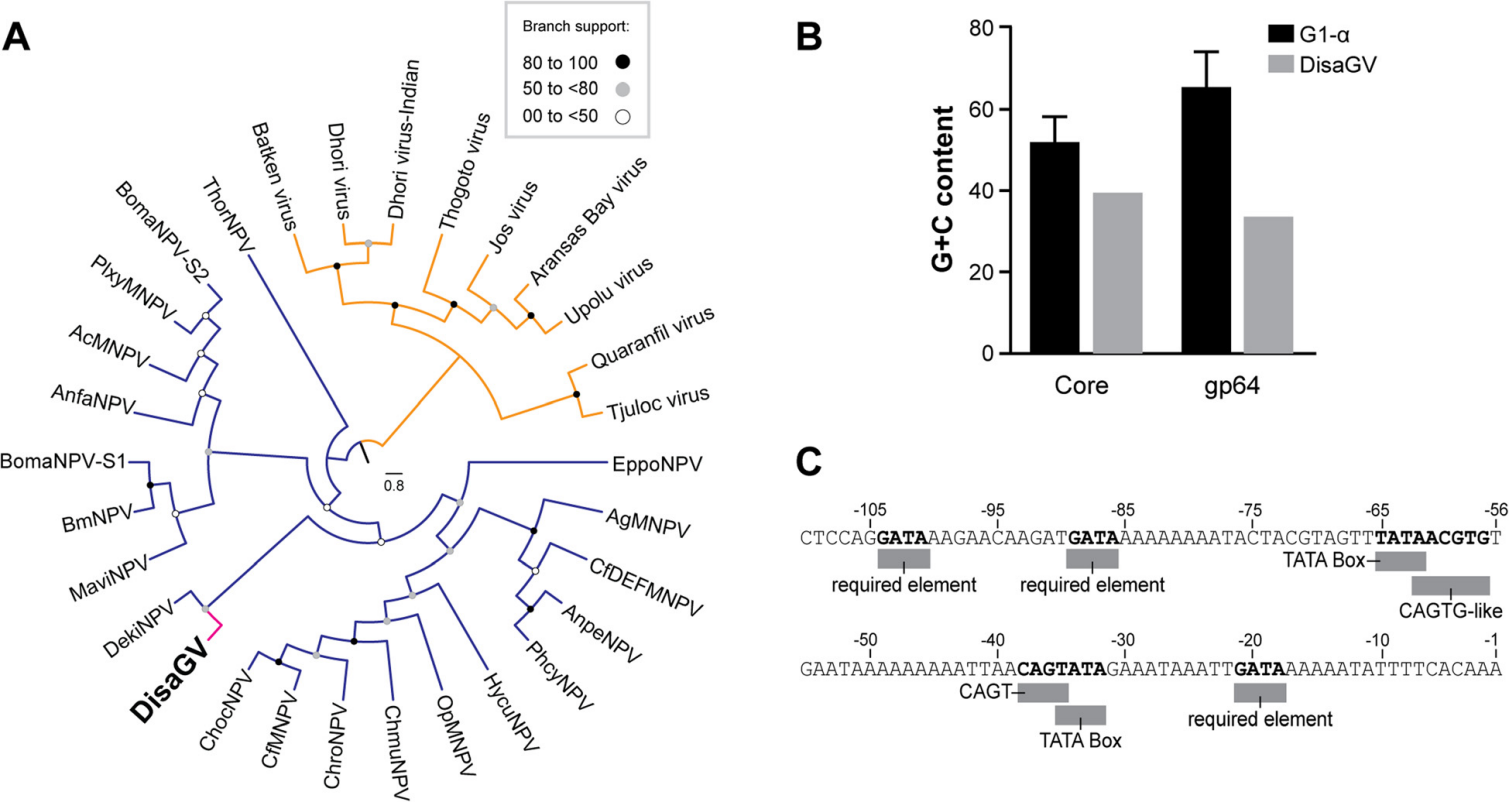
Phylogeny, G + C content and promoter region analyses of the betabaculovirus-encoded *gp64* homolog, *disa118*. **a** The DisaGV homolog is related to DekiNPV. The maximum likelihood (ML) tree was inferred using the predicted amino acid sequence of all the betabaculovirus GP64 (pink), several publicly available Group 1 alphabaculovirus genes (blue), and thogotovirus genes (orange). We performed the RaxML method under the WAG + I + G model with a nonparametric bootstrap for phylogeny reconstruction. Thogotoviruses root the tree that is presented here as a cladogram. **b** Comparison of the G + C content average for the third position of the translational codon in the *gp64* genes from all Group 1 *Alphabaculovirus* (G1-α) and DisaGV. The comparison suggests that *disa118* underwent an adjustment for the low G + C content characteristic of betabaculoviruses. **c** Annotation of 110 bp long from the *disa118* promoter region. The elements and motifs shown were identified based on previously published analyses of alphabaculovirus genomes

The most striking finding in the DisaGV genome was the presence of a *gp64* homolog gene, *disa118*. GP64 is the major envelope fusion protein (EFP) exclusively found in Group I alphabaculoviruses (G1-α) [6]. Both Group 2 alphabaculovirus (G2-α) and betabaculovirus share an analog to the GP64, called F protein, as the major budded virus (BV) EFP [28] which is probably the ancestral EFP in baculovirus [7, 29]. GP64 was acquired probably later by the ancestor of G1-α likely from an insect retrovirus-like element [30, 31] and is clearly related to the glycoprotein found in the genus *Thogotovirus* (from *Orthomyxoviridae*, an ssRNA negative-strand segmented virus family) [32]. Therefore, in attempt to understand both acquisition and evolution of *gp64* into the DisaGV genome, we performed a phylogenetic reconstruction of the gene. We found that DisaGV GP64 clustered with G1-α EFP, suggestive of a horizontal gene transfer (HGT) from G1-α to betabaculovirus (Fig. 6a). Disa-GP64 clustered with *Dendrolimus kikuchii nucleopolyhedrovirus* (DekiNPV). Therefore, *gp64* gene acquisition probably had adaptive value for the ancestor of DisaGV as it had for G1-α. Taken together, these results suggest that the common ancestor of the G1-α acquired this gene once by HGT from some unknown source, which was later transferred to DisaGV or some related ancestral betabaculovirus. Alternatively, the gene was firstly acquired by a DisaGV-related virus and later transferred to the common ancestor of G1-α. An adaptation of *disa118* to the G + C genome content of DisaGV was observed (Fig. 6b) depicting that the gene acquisition is likely not recent [33]. Experimental analysis has shown that the incorporation of GP64 into the genome of *Helicoverpa armigera nucleopolyhedrovirus* (HaNPV), a G2 α-baculovirus, enhanced virus infectivity *in vivo* and *in vitro* [34]. GP64 and F protein can exploit either distinct [35] or similar [36] receptors to entry into host cells. Therefore, *gp64* acquisition has probably enhanced both fusion and binding virus capabilities [37, 38] and possibly replaced functionally the F protein in G1-α [30]. This evolutionary replacement hypothesis is reinforced by the fact that G1-α encode a remnant F protein homolog in their genomes that is unable to compensate for *gp64* loss, albeit probably playing a role in the virus pathogenicity [39]. Interestingly, despite the fact that the DisaGV genome encodes an F protein, large deletions were observed in several reads covering the gene, suggesting existence of genotypes with deleted segments in the sequenced population (data not shown). This feature may indicate that the function of *f protein* has been replaced or complemented by *gp64* in DisaGV. Moreover, we also inspected the 150 nucleotides up-stream from the predicted DisaGV *gp64* ATG start codon and compare with several G1 α-baculovirus *gp64* promoter regions (Fig. 6c). During viral *de novo* synthesis, *gp64* expression is regulated by transcription from both early and late promoters with negative and multiple positive regulatory elements [40]. The *gp64* promoter region size was previously described to be around 140 bp [41–43]. In the DisaGV, we found three required elements GATA (−21, −89, and −104), 2 TATA Box-like (−35 and −76), 2 CACGTG-like (−38 and −61) sequences with mutation on the first C to A in both, and one TATA-box (−35)-associated CAGT (−38). It is noteworthy that TATA-dependent activity and TATA-independent activity is mediated by RNA polymerase II in OpMNPV *gp64* [44], where two of the required GATA and CACGTG specifically bind to host transcription factors and activate transcription from the TATA-dependent *gp64* promoter [44, 45]. The presence of these conserved regulatory expression sequences in the promoter region of *disa-gp64* gene indicates that it must be transcribed and functional. In a recently published work, we found that *disa-gp64* is a functional envelope fusion protein able to replace the native AcMNPV *gp64* in a null recombinant baculovirus [15].

## Conclusion

After structural characterization, complete genome sequence, and phylogenetic analyses of the *Diatraea saccharalis*-infecting virus (DisaGV), we found it to be a novel, distinct lineage of the *Betabaculovirus* genus. The genome seemed to be closely related to CpGV-related group but had the smallest genome found among other betabaculoviruses so far. Its genome encoded a GPCR-like gene and, remarkably for a functional *gp64* gene, which had been previously reported solely for group 1 alphabaculovirus genomes.

## Methods

### Viral origin, confirmation, and electron microscopy

The DisaGV used in this study was obtained from infected larvae *D. saccharalis* collected in the state of Parana, Brazil in 2009. Transmission electron microscopy (TEM) of purified OBs and *granulin* gene amplicon sequence confirmed that the infection was due to a betabaculovirus. The *granulin* amplification was performed with universal primers for the major OB protein gene as previously published [46]. The amplified fragment was purified from an agarose gel after electrophoresis with the GFX® kit (GE Healthcare) following the manufacturer’s instructions, Sanger sequencing reaction was performed with the BigDye kit (Applied Biosystems) and the sequence determined in an automated sequencer ABI Prism® 3100 Genetic Analyzer (Applied Biosystems). For transmission electron microscopy, a suspension of occlusion bodies extracted from larvae infected by DisaGV was prepared, as described elsewhere [10].

### Sequencing system, assembly, and analysis of the DisaGV complete genome

DisaGV genomic DNA was sequenced with the 454 Genome Sequencer (GS) FLX™ Standard (Roche) at the Centro de Genômica de Alto Desempenho do Distrito Federal (Brasília, Brazil). The genome was assembled *de novo* using Geneious 7.0 [47] and confirmed by comparing the restriction endonuclease fragment pattern from digestion of viral DNA to the fragment pattern generated from the assembled sequence. The annotation was performed using Geneious 7.0 to identify the ORFs that started with a methionine codon (ATG) encoding at least 50 amino acids and blastp to identify homologs. Specific primers were designed to amplify and sequence, by Sanger method, all regions in the genome with low coverage (<10 x).

### Phylogenetic analyses and genome comparison

For the *Baculoviridae* phylogeny, a MAFFT alignment [48] was carried out with the concatenated amino acid sequences predicted for the 37 baculovirus core genes. Phylogenetic inference was done using the FastTree method [49], implemented in Geneious. For evaluating putative HGTs events the same alignment method was used for Disa034, Disa038, Disa039 (GPCR-encoding gene), and Disa118 (*gp64* homolog) and the phylogenetic tree was inferred using the RaxML program with 100 replications of non parametric bootstrap [50], implemented in Geneious, with the models WAG + I + G for GP64, WAG + I + G + F for Disa038, WAG + I + F for Disa039, and LG + I + G for Disa034 selected by Prottest 2.4 [51]. The signal peptide and the transmembrane domains were predicted by both the SignalP 4.1 server (http://www.cbs.dtu.dk/services/SignalP/) and the TMHMM Sever v. 2.0 (http://www.cbs.dtu.dk/services/TMHMM/), respectively. Moreover, the complete genome of DisaGV was compared with other betabaculovirus genomes through construction of syntenic maps with the Mauve program in the Geneious 7.1.7 using default parameter settings.

### Availability of supporting data

The datasets of Figs. 3a, 4a, b, 5a and 6a supporting the conclusions of this article are available in the TreeBase [http://treebase.org/treebase-web/home.html] repository, [http://purl.org/phylo/treebase/phylows/study/TB2:S18717].

## Additional files

Additional file 1: Table S1. Gene content. (DOC 313 kb) Additional file 2: Table S2. Virus isolates used in this paper for the reconstruction of the baculovirus phylogeny. (DOC 93 kb)

## Abbreviations

AcMNPV: *Autographa californica multiple nucleopolyhedrovirus*
AdorGV: *Adoxophyes orana granulovirus*
AgMNPV: *Anticarsia gemmatalis multiple nucleopolyhedrovirus*
bp: base pairs
BV: budded virus
CaLGV: *Clostera anastomosis granulovirus*
ChocGV: *Choristoneura occidentalis granulovirus*
ClanGV: *Clostera anachoreta granulovirus*
CpGV: *Cydia pomonella granulovirus*
CrleGV: *Cryptophlebia leucotreta granulovirus*
DekiNPV: *Dendrolimus kikuchii nucleopolyhedrovirus*
DisaGV: *Diatraea saccharalis granulovirus*
EFP: envelope fusion protein
*Egt*: *ecdysteroid UDP-glucosyltransferase*
ErelGV: *Erinnyis ello granulovirus*
G1-α: Group I alphabaculovirus
G2-α: Group II alphabaculovirus
GPCR: G-protein coupled receptor
GV: granulovirus
HaNPV: *Helicoverpa armigera nucleopolyhedrovirus*
HearGV: *Helicoverpa armigera granulovirus*
HGT: horizontal gene transfer
hr: homologous region
LCB: Locally Collinear Block
OB: occlusion body
ORF: open reading frame
PiraGV: *Pieris rapae granulovirus*
PlxyGV: *Plutella xylostella granulovirus*
PsunGV: *Pseudaletia unipuncta granulovirus*
SpliGV-K1: *Spodoptera litura granulovirus* isolate K1
TEM: Transmission electron microscopy
WIV: *Wiseana iridescent virus*
XecnGV: *Xestia c-nigrum granulovirus*
