# Supplementary material for: A betabaculovirus encoding a gp64 homolog
Source: BMC Genomics. 2016 Feb 4;17:94. doi: 10.1186/s12864-016-2408-9 (PMC4741009; doi:10.1186/s12864-016-2408-9)
Supplement: Additional file 1: Table S1. — Gene content. (DOC 313 kb) [file 12864_2016_2408_MOESM1_ESM.doc]

| **Additional file 1: Table S1**. Gene composition and general features of the *Diatraea saccharalis* granulovirus (DisaGV) genome relative to other baculovirus genomes. | | | | | | | | | | |
| --- | --- | --- | --- | --- | --- | --- | --- | --- | --- | --- |
| **ORF** | **Name** | **Position** | **Size**  **(bp)** | **Size**  **(aa)** | **Transcriptional**  **motifs** | **Orthologs - ORF number (identity)** | | | | |
|  |  |  |  |  |  | **AcMNPV** | **CypoGV** | **CrleGV** | **PiraGV** | **ChocGV** |
| **1** | *granulin* | 1 > 747 | 747 | 248 | E, L | 8 (55) | 1 (93) | 1 (91) | 1 (81) | 1 (91) |
| **2** |  | 1084 < 728 | 357 | 118 | E, L | - | 2 (58) | 2 (59) | 2 (51) | 2 (39) |
| **3** | *pk-1* | 1065 > 1877 | 813 | 270 | E, L | 10 (38) | 3 (54) | 3 (58) | 3 (61) | 3 (61) |
| **4** |  | 2112 < 1852 | 261 | 86 | E | - | - | - | - | - |
|  | *hr1* | 1921 - 2134 | 214 | - | - | - | - | - | - | - |
| **5** |  | 2701 < 2132 | 570 | 189 | E, L | - | 4 (51) | 4 (51) | 4 (56) | 5 (49) |
| **6** | *ie-1* | 4218 < 2932 | 1287 | 428 | E | 147 (29) | 7 (45) | 6 (44) | 6 (47) | 7 (41) |
| **7** |  | 4249 > 4818 | 570 | 189 | E, L | 146 (28) | 8 (46) | 7 (47) | 7 (55) | 8 (55) |
| **8** |  | 5134 < 4838 | 297 | 98 | E, L | 145 (38) | 9 (60) | 8 (61) | 8 (65) | 9 (61) |
| **9** | *odv-e18* | 5406 < 5140 | 267 | 88 | E, L | 143 (29) | 14 (76) | 13 (72) | 14 (66) | 12 (73) |
| **10** | *p49* | 6790 < 5393 | 1398 | 465 | E, L | 142 (31) | 15 (58) | 14 (59) | 15 (64) | 13 (60) |
| **11** |  | 7946 < 7368 | 579 | 192 | E, L | - | 16 (52) | 15 (53) | - | - |
| **12** | *odv-e56* | 9070 < 7943 | 1128 | 375 | E | 148 (46) | 18 (70) | 17 (68) | 16 (68) | 14 (72) |
| **13** |  | 9096 > 9299 | 204 | 67 | E | - | - | - | - | - |
| **14** | *pep1* | 9757 < 9260 | 498 | 165 | E, L | - | 20 (57) | 20 (66) | 20 (53) | 17 (70) |
| **15** |  | 9837 > 10406 | 570 | 189 | E, L | - | - | - | - | - |
| **16** | *pep/p10* | 10453 > 11427 | 975 | 324 | E, L | - | 22 (64) | 23 (63) | 22 (60) | 18 (64) |
| **17** | *pep2* | 11439 > 11873 | 435 | 144 | E, L | - | 23 (71) | 24 (70) | 22 (70) | 19 (66) |
| **18** |  | 12838 < 11885 | 954 | 317 | E, L | - | 29 (36) | - | 24 (27) | 22 (29) |
| **19** |  | 13103 < 12879 | 225 | 74 | E, L | - | - | - | - | - |
| **20** |  | 13528 > 14025 | 498 | 165 | E, L | - | - | - | - | - |
| **21** | *gp41* | 14904 < 14047 | 858 | 285 | E, L | 80 (32) | 104 (67) | 95 (69) | 88 (71) | 83 (66) |
| **22** |  | 15445 < 14849 | 597 | 198 | E, L | 81 (47) | 103 (73) | 94 (71) | 87 (71) | 82 (70) |
| **23** |  | 15740 < 15429 | 312 | 103 | E | 82 (26) | 102 (47) | 93 (43) | 86 (47) | 81 (45) |
| **24** | *vp91* | 15718 > 17355 | 1638 | 545 | E, L | 83 (26) | 101 (55) | 92 (55) | 89 (53) | 80 (53) |
| **25** | *efp/f protein* | 17418 > 19028 | 1611 | 536 | E, L | 23 (22) | 31 (38) | 30 (39) | 26 (40) | 23 (41) |
| **26** |  | 19145 > 19630 | 486 | 161 | E, L | - | - | - | - | - |
| **27** |  | 19829 < 19587 | 243 | 80 | E, L | - | - | - | - | - |
| **28** |  | 19804 > 19989 | 186 | 61 | - | - | - | - | - | - |
| **29** |  | 20609 < 20007 | 603 | 200 | E, L | - | 33 (38) | 32 (41) | 28 (42) | 24 (38) |
| **30** | *pif-3* | 20637 > 21194 | 558 | 185 | E, L | 115 (39) | 35 (53) | 34 (48) | 30 (52) | 26 (50) |
| **31** | *odv-e66* | 23470 < 21185 | 2286 | 761 | L | 46 (58) | 37 (65) | 35 (67) | 45 (56) | 27 (60) |
| **32** |  | 23509 > 23820 | 312 | 103 | E, L | - | 39 (66) | 36 (68) | 31 (64) | 28 (55) |
| **33** |  | 24140 < 23856 | 285 | 94 | E, L | - | - | - | - | - |
| **34** |  | 25142 < 24210 | 933 | 310 | E | - | - | - | - | - |
| **35** |  | 25067 > 25219 | 153 | 50 | E | - | - | - | - | - |
| **36** | *lef-2* | 25278 > 25796 | 519 | 172 | E | 6 (27) | 41 (48) | 38 (49) | 33 (54) | 29 (51) |
| **37** |  | 25783 > 26028 | 246 | 81 | E, L | - | 42 (33) | 39 (37) | 34 (48) | 30 (41) |
| **38** |  | 26098 > 27294 | 1197 | 398 | E, L | - | - | - | - | - |
| **39** |  | 27632 < 27291 | 342 | 113 | E | - | - | - | - | - |
| **40** | *metalloproteinase* | 28746 < 27634 | 1113 | 370 | E, L | - | 46 (36) | 43 (40) | 37 (40) | 33 (39) |
| **41** | *p13* | 28756 > 29556 | 801 | 266 | E, L | - | 47 (59) | 44 (61) | 38 (54) | 34 (60) |
|  | *hr-2* | 29572 - 29929 | 358 | - | - | - | - | - | - | - |
| **42** | *pif-2* | 30025 > 31146 | 1122 | 373 | E, L | 22 (52) | 48 (70) | 45 (71) | 40 (70) | 35 (66) |
| **43** |  | 31209 > 31592 | 384 | 127 | E | - | - | - | - | - |
| **44** |  | 31794 < 31597 | 198 | 65 | E, L | - | 49 (43) | 46 (35) | 41 (43) | - |
| **45** |  | 31814 > 33667 | 1854 | 617 | E, L | - | 50 (46) | 47 (51) | 42 (31) | - |
| **46** |  | 34271 < 33672 | 600 | 199 | E, L | 106 (39) | 52 (68) | 50 (71) | 43 (72) | 37 (72) |
| **47** |  | 34283 > 34432 | 150 | 49 | E, L | 110 (24) | 53 (67) | 51 (63) | 44 (76) | 38 (81) |
| **48** | *v-ubq* | 34775 < 34419 | 357 | 118 | E | 35 (79) | 54 (82) | 52 (84) | 45 (82) | 39 (85) |
| **49** | *odv-ec43* | 34779 > 35816 | 1038 | 345 | E, L | 109 (32) | 55 (56) | 53 (58) | 46 (69) | 40 (65) |
| **50** |  | 35822 > 36016 | 195 | 64 | E, L | - | 56 (47) | 54 (56) | 47 (45) | 41 (58) |
| **51** | *39k/pp31* | 36755 < 36018 | 738 | 245 | E, L | 36 (35) | 57 (46) | 52 (44) | 48 (51) | 55 (41) |
| **52** | *lef-11* | 37026 < 36745 | 282 | 93 | E, L | 37 (27) | 58 (66) | 53 (64) | 49 (59) | 56 (56) |
| **53** | *p74* | 39004 < 36950 | 2055 | 684 | E, L | 138 (42) | 60 (60) | 58 (61) | 51 (60) | 46 (58) |
| **54** |  | 39497 < 39057 | 441 | 146 | E, L | - | - | - | - | - |
| **55** | *acetyltransferase* | 40081 < 39497 | 585 | 194 | E, L | - | - | - | 56 (66) | 48 (58) |
| **56** |  | 40513 < 40094 | 420 | 139 | E, L | - | 62 (70) | 60 (58) | 55 (47) | 49 (86) |
|  | *hr-3* | 40146 - 40318 | 173 | - | - | - | - | - | - | - |
| **57** | *p47* | 40557 > 41711 | 1155 | 384 | E, L | 40 (42) | 68 (65) | 61 (65) | 56 (66) | 50 (66) |
| **58** | *bv-e31* | 41746 > 42399 | 654 | 217 | E, L | 38 (42) | 69 (76) | 62 (75) | 57 (76) | 51 (72) |
|  | *hr-4* | 42416 - 43074 | 659 | - | - | - | - | - | - | - |
| **59** |  | 42631 < 42455 | 177 | 58 | - | - | - | - | - | - |
| **60** | *p24* | 43135 > 43626 | 492 | 163 | E, L | 129 (32) | 71 (61) | 63 (64) | 58 (56) | 52 (63) |
| **61** | *38.8k* | 44072 < 43647 | 426 | 141 | - | 13 (30) | 73 (35) | 65 (40) | 62 (45) | 54 (37) |
| **62** | *lef-1* | 44760 < 44053 | 708 | 235 | E | 14 (31) | 74 (59) | 66 (59) | 60 (63) | 55 (59) |
| **63** | *pif-1* | 44770 > 46323 | 1554 | 517 | E, L | 119 (36) | 75 (60) | 67 (60) | 61 (58) | 56 (59) |
| **64** |  | 46328 > 46690 | 363 | 120 | E, L | - | 70 (34) | - | - | - |
| **65** | *iap-3* | 46790 > 47584 | 795 | 264 | E, L | 27 (32) | 17 (54) | 16 (48) | - | 84 (50) |
| **66** |  | 47630 > 47782 | 153 | 51 | E, L | - | - | - | - | - |
| **67** |  | 47800 > 48003 | 204 | 67 | E, L | 150 (22) | 79 (35) | 70 (35) | - | 59 (38) |
| **68** | *lef-6* | 48269 < 47985 | 285 | 94 | E | 28 (31) | 80 (45) | 71 (43) | 65 (58) | 60 (52) |
| **69** | *dbp* | 49084 < 48287 | 798 | 265 | E, L | 25 (22) | 81 (46) | 72 (44) | 66 (50) | 61 (48) |
| **70** |  | 49321 < 49103 | 219 | 72 | E, L | - | 82 (51) | 73 (46) | 70 (60) | 62 (48) |
| **71** |  | 49847 < 49263 | 585 | 194 | E, L | - | 82 (27) | 73 (27) | 67 (41) | 63 (34) |
| **72** | *p48/p45* | 49869 > 51041 | 1173 | 390 | E, L | 103 (33) | 83 (70) | 74 (68) | 68 (73) | 64 (69) |
| **73** |  | 51068 > 51355 | 288 | 95 | E, L | 102 (26) | 84 (50) | 75 (49) | 69 (50) | 65 (40) |
| **74** |  | 52171 < 51395 | 777 | 258 | E, L | - | - | - | - | - |
| **75** | *odv-c42/p40* | 52403 > 53563 | 1161 | 386 | E, L | 101 (22) | 85 (59) | 76 (58) | 70 (57) | 66 (55) |
| **76** | *p6.9* | 53571 > 53756 | 186 | 61 | E, L | - | - | - | - | - |
| **77** | *lef-5* | 54493 < 53792 | 702 | 234 | E, L | 99 (42) | 87 (68) | 78 (68) | 72 (71) | 68 (66) |
| **78** | *38 k* | 54443 > 55354 | 912 | 303 | E, L | 98 (39) | 88 (60) | 79 (59) | 73 (73) | 69 (66) |
| **79** | *dut* | 55338 > 55808 | 471 | 156 | E, L | - | - | - | - | - |
| **80** |  | 55805 > 56179 | 375 | 124 | - | - | - | - | - | - |
| **81** | *odv-e28/pif-4* | 56684 < 56199 | 486 | 161 | E, L | 96 (35) | 89 (62) | 80 (64) | 74 (61) | 70 (55) |
| **82** | *helicase-1* | 56668 > 60051 | 3384 | 1127 | E, L | 95 (26) | 90 (52) | 81 (52) | 75 (57) | 71 (54) |
| **83** | *odv-e25* | 60707 < 60069 | 639 | 212 | E, L | 94 (37) | 91 (69) | 82 (69) | 76 (68) | 72 (70) |
| **84** | *p18* | 61209 < 60727 | 483 | 160 | E, L | 93 (33) | 92 (44) | 83 (40) | 77 (49) | 73 (46) |
| **85** | *sox/p33* | 61224 > 61979 | 756 | 251 | E, L | 92 (36) | 93 (66) | 84 (64) | 78 (67) | 74 (66) |
| **86** | *lef-4* | 63298 < 61976 | 1323 | 440 | E, L | 90 (32) | 95 (54) | 86 (52) | 80 (57) | 75 (55) |
| **87** | *vp39* | 63312 > 64166 | 855 | 284 | E, L | 89 (33) | 96 (60) | 87 (62) | 81 (63) | 76 (61) |
| **88** | *odv-ec27* | 64217 > 65029 | 813 | 270 | L | 144 (31) | 97 (61) | 88 (61) | 82 (64) | 77 (55) |
|  | *hr5* | 65061 - 65488 | 428 | - | - | - | - | - | - | - |
| **89** |  | 65127 > 65417 | 291 | 96 | E | - | - | - | - | - |
| **90** |  | 66499 < 65465 | 1035 | 344 | E, L | - | 99 (35) | 90 (35) | 83 (36) | 78 (37) |
| **91** |  | 66528 > 66722 | 195 | 64 | E, L | - | 100 (50) | 91 (51) | 84 (50) | 79 (60) |
| **92** |  | 66729 > 66995 | 267 | 88 | E, L | 78 (42) | 105 (41) | 96 (46) | 89 (45) | 85 (48) |
| **93** | *vlf-1* | 66940 > 68055 | 1116 | 371 | E, L | 77 (34) | 106 (73) | 97 (74) | 90 (77) | 86 (67) |
| **94** |  | 68079 > 68330 | 252 | 83 | E, L | 76 (26) | 107 (67) | 98 (65) | 91 (70) | 88 (68) |
| **95** |  | 68345 > 68800 | 456 | 151 | E, L | 75 (28) | 108 (57) | 99 (58) | 92 (63) | 89 (63) |
| **96** |  | 69174 < 68830 | 345 | 114 | E, L | - | 110 (26) | 100 (23) | - | - |
| **97** | *dna pol* | 72280 < 69203 | 3078 | 1025 | E | 65 (33) | 111(65) | 101(66) | 93 (68) | 90 (66) |
| **98** | *desmoplakin* | 72255 > 73895 | 1641 | 546 | E, L | 66 (27) | 112 (31) | 102 (32) | 98 (32) | 91 (33) |
|  | *hr-6* | 73837 - 74251 | 415 | - | - | - | - | - | - | - |
| **99** | *lef-3* | 75316 < 74249 | 1068 | 355 | E, L | 67 (24) | 113 (35) | 103 (38) | 95 (45) | 92 (43) |
| **100** | *odv-nc42* | 75282 > 75668 | 387 | 128 | E, L | 68 (34) | 114 (65) | 104 (57) | 96 (65) | 93 (62) |
| **101** |  | 75849 < 75661 | 189 | 62 | E | - | - | - | - | - |
| **102** |  | 75766 > 76248 | 483 | 160 | E, L | - | 115 (38) | 105 (33) | 97 (39) | 94 (33) |
| **103** | *iap-5* | 76307 > 77101 | 795 | 264 | E | - | 116 (63) | 106 (60) | 98 (58) | 95 (58) |
| **104** | *lef-9* | 77106 > 78584 | 1479 | 492 | L | 62 (53) | 117 (73) | 107 (72) | 99 (75) | 96 (73) |
| **105** | *fp25k* | 78590 > 79030 | 441 | 146 | E, L | 61 (36) | 118 (68) | 108 (66) | 100 (72) | 97 (67) |
| **106** |  | 80190 < 79057 | 1134 | 377 | E | - | - | - | - | - |
| **107** |  | 80444 < 80193 | 252 | 83 | E | - | - | - | - | - |
| **108** |  | 82066 < 80555 | 1512 | 503 | E | - | - | - | - | - |
| **109** | *dna ligase* | 83743 < 82106 | 1638 | 545 | E, L | - | 120 (59) | 110 (59) | 102 (59) | 99 (58) |
| **110** |  | 83942 < 83745 | 198 | 65 | E, L | - | - | - | - | - |
| **111** |  | 84027 > 84341 | 315 | 104 | E, L | - | 124 (55) | 114 (56) | 106 (49) | 103 (55) |
| **112** |  | 84499 < 84317 | 183 | 60 | E | - | - | - | - | - |
| **113** | *alk-exo* | 84414 > 85610 | 1197 | 398 | E, L | 133 (33) | 125 (53) | 115(53) | 107 (64) | 104 (56) |
| **114** | *helicase-2* | 85513 > 86805 | 1293 | 430 | E, L | - | 126 (59) | 116 (54) | 108 (59) | 105 (52) |
| **115** | *rr1* | 88666 < 86849 | 1818 | 605 | E | - | 127 (54) | - | - | - |
| **116** | *rr2a* | 88765 > 89892 | 1128 | 375 | L | - | 128 (59) | - | - | - |
| **117** |  | 89960 < 89808 | 153 | 50 | E | - | - | - | - | - |
| **118** | *gp64* | 89952 > 91469 | 1518 | 505 | E, L | 128 (74) | - | - | - | - |
| **119** | *lef-8* | 93976 < 91472 | 2505 | 834 | E, L | 50 (48) | 131 (69) | 119 (67) | 110 (70) | 107 (68) |
| **120** |  | 94000 > 94404 | 405 | 134 | E, L | 53 (36) | 134 (67) | 121 (69) | 113 (68) | 109 (63) |
|  | *hr-7* | 94399 - 94911 | 513 | - | - | - | - | - | - | - |
| **121** |  | 95772 < 94963 | 810 | 269 | E, L | - | 135 (41) | 122 (29) | 114 (38) | 110 (31) |
| **122** |  | 96139 < 95942 | 198 | 65 | E, L | - | 136 (41) | 123 (44) | 115 (50) | 111 (44) |
| **123** | *lef-10* | 96120 > 96353 | 234 | 77 | E, L | 53a (38) | 137 (52) | 124 (56) | 120 (61) | 112 (55) |
| **124** | *vp1054* | 96214 > 97197 | 984 | 327 | E, L | 54 (30) | 138 (59) | 125 (58) | 116 (66) | 113 (59) |
| **125** | *me53* | 97422 > 98366 | 945 | 314 | E | 139 (27) | 143 (52) | 129 (47) | 125 (52) | 116 (46) |
| **Note**: Position, transcriptional orientation and length (bp and aa) of 125 putative ORFs of the DisaGV genome. The ORFs were compared with their respective homologs in AcMNPV and 4 betabaculoviruses in terms of corresponding ORF number and amino acid identity (ID %). DisaGV unique ORFs are shown in red, betabaculovirus-specific ORFs in green, ORFs conserved in all baculovirus genomes (core genes) in blue. The conserved early (E; TATAW, TATAWTW e/ou TATAWAW) and late (L; A/T/GTAAG) transcriptional motifs within 450 bp upstream each putative ORF are also shown. | | | | | | | | | | |
