# Supplementary material for: A betabaculovirus encoding a gp64 homolog
Source: BMC Genomics. 2016 Feb 4;17:94. doi: 10.1186/s12864-016-2408-9 (PMC4741009; doi:10.1186/s12864-016-2408-9)
Supplement: Additional file 2: Table S2. — Virus isolates used in this paper for the reconstruction of the baculovirus phylogeny. (DOC 93 kb) [file 12864_2016_2408_MOESM2_ESM.doc]

| **Additional file 2: Table S2**. List of virus isolates used in this paper for the reconstruction of the baculovirus phylogeny in the FIG. 3A. The species from the genera *Alphabaculovirus* (dark blue), *Betabaculovirus* (pink), *Gammabaculovirus* (orange), and *Deltabaculovirus* (light blue) are presented here together with the abbreviation used in the main text, the host family where the virus was isolated from, and the Genbank accession number as well. | | | |
| --- | --- | --- | --- |
| **Species** | **Abbreviation** | **Host family** | **Accession** |
| Adoxophyes honmai nucleopolyhedrovirus | AdhoNPV | Tortricidae | AP006270 |
| Adoxophyes orana nucleopolyhedrovirus | AdorNPV | Tortricidae | EU591746 |
| Agrotis ipsilon multiple nucleopolyhedrovirus strain illinois | AgipMNPV | Noctuidae | EU839994 |
| Agrotis segetum nucleopolyhedrovirus | AgseNPV | Noctuidae | DQ123841 |
| Apocheima cinerarium nucleopolyhedrovirus | ApciNPV | Geometridae | FJ914221 |
| Buzura suppressaria nucleopolyhedrovirus | BusuNPV | Geometridae | KF611977 |
| Chrysodeixis chalcites nucleopolyhedrovirus | ChchNPV | Noctuidae | AY864330 |
| Clanis bilineata nucleopolyhedrovirus | ClbiNPV | Sphingidae | DQ504428 |
| Ectropis obliqua nucleopolyhedrovirus strain A1 | EcobNPV-A1 | Geometridae | DQ837165 |
| Euproctis pseudoconspersa nucleopolyhedrovirus | EupsNPV | Lymantriidae | FJ227128 |
| Helicoverpa armigera multiple nucleopolyhedrovirus | HaMNPV | Noctuidae | EU730893 |
| Helicoverpa armigera nucleopolyhedrovirus C1 | HaNPV-C1 | Noctuidae | AF303045 |
| Helicoverpa zea single nucleopolyhedrovirus USA | HzSNPV-USA | Noctuidae | AF334030 |
| Hemileuca sp. nucleopolyhedrovirus | HespNPV | Saturniidae | KF158713 |
| Lambdina fiscellaria nucleopolyhedrovirus | LafiNPV | Geometriidae | KP752043 |
| Leucania separata nuclear polyhedrovirus strain AH1 | LeseNPV | Noctuidae | AY394490 |
| Lymantria díspar multiple nucleopolyhedrovirus | LdMNPV | Lymantriidae | AF081810 |
| Lymantria xylina multiple nucleopolyhedrovirus | LyxyMNPV | Lymantriidae | GQ202541 |
| Mamestra brassicae multiple nucleopolyhedrovirus strain Chb1 | MbMNPV-CHb1 | Noctuidae | JX138237 |
| Mamestra configurata nucleopolyhedrovirus-A strain 90/2 | MacoNPV-A 90/2 | Noctuidae | U59461 |
| Mamestra configurata nucleopolyhedrovirus B | MacoNPV-B | Noctuidae | AY126275 |
| Orgyia leucostigma nucleopolyhedrovirus isolate CFS-77 | OrleNPV | Lymantriidae | EU309041 |
| Peridroma sp. nucleopolyhedrovirus | PespNPV | Noctuidae | KM009991 |
| Perigonia lusca single nucleopolyhedrovirus | PeluSNPV | Sphigidae | KM596836 |
| Pseudoplusia includens single nucleopolyhedrovirus IE | PsinSNPV | Noctuidae | KJ631622 |
| Spodoptera exigua nucleopolyhedrovirus | SeMNPV | Noctuidae | AF169823 |
| Spodoptera frugiperda multiple nucleopolyhedrovirus isolate 19 | SfMNPV-19 | Noctuidae | EU258200 |
| Spodoptera litoralis nucleopolyhedrovirus isolate AN1956 | SpliNPV-1956 | Noctuidae | JX454574 |
| Spodoptera litura nucleopolyhedrovirus G2 | SpliNPV-G2 | Noctuidae | AF325155 |
| Spodoptera litura nucleopolyhedrovirus II | SpliNPV-II | Noctuidae | EU780426 |
| Sucra jujuba nucleopolyhedrovirus | SujuNPV | Geometridae | KJ676450 |
| Trichoplusia ni single nucleopolyhedrovirus | TnSNPV | Noctuidae | DQ017380 |
| Autographa californica nucleopolyhedrovirus clone C6 | AcMNPV-C6 | Noctuidae | L22858 |
| Anticarsia gemmatalis nucleopolyhedrovirus | AgMNPV | Noctuidae | DQ813662 |
| Antheraea pernyi nucleopolyhedrovirus isolate L2 | AnpeNPV-L2 | Saturniidae | EF207986 |
| Bombyx mori nucleopolyhedrovirus strain T3 | BmNPV-T3 | Bombycidae | L33180 |
| Bombyx mandarina nucleopolyhedrovirus S2 | BomaNPV-S2 | Bombycidae | JQ071499 |
| Choristoneura fumiferana defective multiple nucleopolyhedrovirus | CfDEFMNPV | Tortricidae | AY327402 |
| Choristoneura fumiferana multiple nucleopolyhedrovirus | CfMNPV | Tortricidae | AF512031 |
| Choristoneura murinana nucleopolyhedrovirus | ChmuNPV | Tortricidae | KF894742 |
| Choristoneura occidentalis nucleopolyhedrovirus | ChocNPV | Tortricidae | KC961303 |
| Choristoneura rosaceana nucleopolyhedrovirus | ChroNPV | Tortricidae | KC961304 |
| Condylorrhiza vestigialis multiple nucleopolyhedrovirus | CoveMNPV | Crambidae | KJ631623 |
| Dendrolimus kikuchii nucleopolyhedrovirus | DekiNPV | Lasiocampidae | JX193905 |
| Epiphyas postvittana nucleopolyhedrovirus | EppoNPV | Tortricidae | AY043265 |
| Hyphantria cunea nucleopolyhedrovirus | HycuNPV | Arctiidae | AP009046 |
| Maruca vitrata multiple nucleopolyhedrovirus | MaviMNPV | Crambidae | EF125867 |
| Orgyia pseudotsugata multiple nucleopolyhedrovirus | OpMNPV | Lymantriidae | U75930 |
| Philosamia cynthia ricini nucleopolyhedrovirus | PhcyNPV | Saturniidae | JX404026 |
| Plutella xylostella multiple nucleopolyhedrovirus isolate CL3 | PlxyMNPV | Plutellidae | DQ457003 |
| Rachiplusia ou multiple nucleopolyhedrovirus | RoMNPV | Noctuidae | AY145471 |
| Thysanoplusia orichalcea nucleopolyhedrovirus | ThorNPV | Noctuidae | JX467702 |
| Adoxophyes orana granulovirus | AdorGV | Tortricidae | AF547984 |
| Agrotis segetum granulovirus-L1 | AgseGV-L1 | Noctuidae | KC994902 |
| Choristoneura occidentalis granulovirus | ChocGV | Tortricidae | DQ333351 |
| Clostera anastomosis granulovirus | CaLGV | Notodontidae | KC179784 |
| Clostera anachoreta granulovirus | ClanGV | Notodontidae | HQ116624 |
| Clostera anastomosis granulovirus Strain B | ClanGV-B | Notodontidae | KR091910 |
| Cryptophlebia leucotreta granulovirus isolate CV3 | CrleGV | Tortricidae | AY229987 |
| Cydia pomonella granulovirus | CpGV | Tortricidae | U53466 |
| Diatraea saccharalis granulovirus | **DisaGV** | **Crambidae** | **KP296186** |
| Epinotia aporema granulovirus | EpapGV | Tortricidae | JN408834 |
| Erinnyis ello granulovirus | ErelGV | Sphingidae | KJ406702 |
| Helicoverpa armigera granulovirus | HaGV | Noctuidae | EU255577 |
| Phthorimaea operculella granulovirus | PhopGV | Gelechiidae | AF499596 |
| Pieris rapae granulovirus E3 | PiraGV-E3 | Pieridae | GU111736 |
| Plutella xylostella granulovirus | PlxyGV | Plutellidae | AF270937 |
| Pseudaletia unipuncta granulovirus | PsunGV-Hawaiin | Noctuidae | EU678671 |
| Spodoptera frugiperda granulovirus | SpfrGV | Noctuidae | KM371112 |
| Spodoptera litura granulovirus isolate K1 | SpliGV | Noctuidae | DQ288858 |
| Xestia c-nigrum granulovirus | XcGV | Noctuidae | AF162221 |
| Neodiprion sertifer nucleopolyhedrovirus | NeseNPV | Diprionidae | AY430810 |
| Neodiprion lecontei nucleopolyhedrovirus | NeleNPV | Diprionidae | AY349019 |
| Neodiprion abietis nucleopolyhedrovirus | NeabNPV | Diprionidae | DQ317692 |
| Culex nigripalpus nucleopolyhedrovirus | CuniNPV | Culicidae | AF403738 |
